# Beyond the ventricle: the atrial contribution to feline hypertrophic cardiomyopathy and implications for diagnosis, prognosis, and emerging therapy

**DOI:** 10.3389/fvets.2026.1979805

**Published:** 2026-10-28

**Authors:** Sumade Kongchatree, Jatuporn Rattanasrisomporn

**Affiliations:** Department of Companion Animal Clinical Sciences, Faculty of Veterinary Medicine, Kasetsart University, Bangkok, Thailand

**Keywords:** arterial thromboembolism, atrial cardiomyopathy, companion animal, comparative medicine, feline hypertrophic cardiomyopathy, left atrial function, sirolimus, speckle-tracking echocardiography

## Abstract

Hypertrophic cardiomyopathy is the most common heart disease of cats and is conventionally characterised by its ventricular phenotype. The clinical events that determine quality and duration of life in affected cats, however, are atrial in their proximate pathophysiology. Congestive heart failure arises from elevated left atrial pressure transmitted to the pulmonary venous bed, arterial thromboembolism arises from blood stasis in a remodelled and dysfunctional left atrial appendage, and survival tracks left atrial size and function independently of left ventricular wall thickness. Recent longitudinal evidence indicates that lower left atrial fractional shortening can be detected before the diagnostic threshold for left ventricular hypertrophy is met, suggesting the atrium registers the disease before the ventricular wall-thickness criterion does. Despite this body of evidence, contemporary diagnostic frameworks, prognostic models, and the regulatory endpoint underpinning the recent conditional approval of delayed-release rapamycin remain anchored on the ventricle. This narrative review examines the atrial contribution to feline hypertrophic cardiomyopathy through five themes: atrial pathobiology, atrial imaging, atrial-driven outcomes, the implications for emerging disease-modifying therapy, and the practical implications for the cardiology consultation. We outline a framework for stage-anchored therapy decisions and a research agenda prioritising atrial trial endpoints and multicentre replication. In a disease affecting 14.7% of apparently healthy cats, with an 11.3% 10-year thromboembolic risk in preclinical cohorts, we propose that the atrium should now be at the centre of feline hypertrophic cardiomyopathy rather than at its periphery.

## 1 Introduction

In a longitudinal cohort of cats from rehoming centres followed for a median of 5.6 years, an independent predictor of later development of hypertrophic cardiomyopathy was not a measure of the left ventricle. It was lower left atrial fractional shortening at baseline. Of 107 cats followed, 19 developed HCM. A baseline left atrial fractional shortening at or below 25% was independently associated with that outcome on multivariable Cox proportional hazards analysis, and cats whose baseline value exceeded 25% were reported to be 91% less likely to develop the phenotype. The multivariable hazard ratios were presented graphically rather than tabulated, and the estimate rests on only 19 events (1). The cats whose left atrial fractional shortening was already reduced, while their ventricular wall thicknesses were normal or equivocal, were the cats who went on to develop overt hypertrophic cardiomyopathy. The same temporal pattern has been demonstrated in humans. Sarcomere mutation carriers without left ventricular hypertrophy show measurable abnormalities of left atrial structure and function on cardiac magnetic resonance imaging (2). The two designs converge without being parallel: the human carriers were genotype-positive and phenotype-negative, whereas the CatScan II cats were phenotypically equivocal and not genotyped. In two species, with different methods and different observers, atrial dysfunction was detectable before ventricular hypertrophy appeared. Whether this reflects truly upstream atrial pathology, or a disease already present that the wall-thickness criterion has not yet registered, is examined in Section 2. On either reading, a framework anchored to wall thickness is systematically late to the disease it is intended to detect. The atrium is not merely a downstream consequence of feline hypertrophic cardiomyopathy. It may be an upstream signal of it.

Hypertrophic cardiomyopathy (HCM) is the most common heart disease of cats. It affects 14.7% (95% CI 12.3–17.4%) of apparently healthy cats screened in rehoming centres, with a case definition of LV wall thickness of 6 mm or more and with hypertensive and hyperthyroid cats excluded (3). That prevalence estimate is itself generated by the wall-thickness criterion that this review argues registers the disease late. In humans, the disease is conventionally characterised by its ventricular phenotype: concentric, often asymmetric thickening of the left ventricular myocardium in the absence of an alternative explanation (4). Feline HCM has been understood through the same conceptual frame. Yet the clinical events that determine quality and duration of life in affected cats are atrial events in their proximate pathophysiology: congestive heart failure (CHF), arterial thromboembolism (ATE), and cardiac death. CHF arises from elevated left atrial pressure transmitted to the pulmonary venous bed. ATE arises from blood stasis in a remodelled, dysfunctional left atrial appendage. Left atrial size and function are retained as independent predictors of survival in multivariable models (5–8). Severe LA enlargement remains the strongest single echocardiographic predictor of imminent CHF and ATE (9).

The field has noticed the atrium. The 2020 ACVIM consensus statement on feline cardiomyopathies places left atrial size at the centre of the B1/B2 staging distinction precisely because it predicts adverse outcomes (9). Recent imaging studies have extended atrial assessment from chamber size to atrial mechanics, including fractional shortening, ejection fraction, peak atrial longitudinal strain, and atrial stiffness (7, 10, 11). What the field has done is more subtle and, in the disease-modifying era, more consequential. It has positioned the atrium as a prognostic marker of a disease that is fundamentally ventricular, rather than as a structure whose pathobiology is itself the disease’s clinical engine. This is a conceptual frame, not an empirical one. Yet conceptual frames shape what is measured, what is reported, what is approved, and what is taught.

That distinction was clinically tolerable when feline HCM had no disease-modifying therapy. The 2025 conditional approval of delayed-release rapamycin (Felycin-CA1, sirolimus) for management of ventricular hypertrophy in cats with subclinical HCM (12, 13) makes the distinction consequential. The pivotal RAPACAT trial reported that low-dose delayed-release rapamycin over 180 days significantly slowed progression of maximum left ventricular wall thickness relative to placebo. The regulatory indication followed that endpoint: ventricular hypertrophy (13). Whether mTOR inhibition alters atrial remodelling, atrial fibrosis, atrial mechanics, or the atrial-driven outcomes of CHF and ATE is not addressed by the available trial data. Veterinary cardiology now has a disease-modifying drug whose evidentiary basis sits one step removed from the events the disease causes.

This narrative review proposes that this conceptual asymmetry is no longer tenable. We synthesise the case for an atrial-centric framework, organised around five themes: atrial pathobiology, atrial imaging, atrial-driven outcomes, the implications for disease-modifying therapy, and the practical implications for the cardiology consultation. We then propose a research and practice agenda. The intent is not to displace the ventricle from feline HCM. It is to give the atrium the position that the disease’s clinical reality already gives it.

### 1.1 Scope and literature search

This review is restricted to the left atrium except where explicitly noted (Section 2.5). Literature was identified through searches of PubMed and Google Scholar to August 2026. Search terms were combinations of feline, cat, hypertrophic cardiomyopathy, left atrium, atrial, strain, speckle tracking, stiffness, thromboembolism, rapamycin, and sirolimus, supplemented by hand-searching of reference lists. Selection reflected the authors’ judgement of relevance rather than systematic criteria.

## 2 Atrial pathobiology in feline HCM

### 2.1 The haemodynamic cascade

The pathobiologic sequence in feline HCM begins in the left ventricle and ends in the atrium, but the atrium is more than a passive endpoint of that sequence. Concentric ventricular hypertrophy, with its associated derangements of myocyte architecture, interstitial fibrosis, and microvascular remodelling, produces diastolic dysfunction: impaired ventricular relaxation and reduced compliance (8). To maintain stroke volume against a stiffening ventricle, left atrial pressure must rise. Chronic exposure of the atrial myocardium to elevated filling pressures imposes a load the atrium was not built to sustain indefinitely (14, 15). The atrium responds, as the ventricle does, with structural and functional remodelling. But the atrium’s remodelling repertoire is narrower. Its ability to compensate is limited by its thin wall, its lower mass, and its structural role as a reservoir-conduit rather than as a pump.

### 2.2 Structural remodelling: dilation, fibrosis, and atrial cardiomyopathy

The most readily observed structural consequence of chronic atrial pressure elevation is dilation. The finding is so consistent in feline HCM that left atrial size has anchored prognostic stratification for two decades (5, 7, 9). Dilation, however, is the gross expression of changes that begin at the tissue level. The histopathologic hallmarks of HCM (interstitial fibrosis, myocyte hypertrophy, and myocyte disarray) have been characterised principally in the ventricular myocardium of affected cats (16), and systematic characterisation of the feline atrial myocardium remains sparse. In humans, contemporary imaging and electrophysiology studies have led to the formalisation of an “atrial cardiomyopathy” or “atrial myopathy” construct. In this construct the atrium is itself a participant in the disease process rather than a downstream victim, with implications for thromboembolism risk, atrial fibrillation susceptibility, and contractile reserve (17). An analogous frame has not been formally adopted in veterinary cardiology. The imaging substrate that supports it is observable in cats, while the histopathologic substrate is established in humans and remains uncharacterised in the feline atrium. The atrium in feline HCM does not merely enlarge; it remodels. The claim rests on three legs: elevated atrial stiffness in affected cats (11), atrial functional impairment demonstrable independently of atrial size (7), and the human histopathologic substrate as cross-species analogy (17). It is, at present, an imaging-inferred case. A feline genotype-positive population allows the temporal question to be examined directly. Maine Coon cats carrying the MYBPC3 A31P mutation show systolic and diastolic ventricular myocardial functional abnormality on tissue Doppler imaging before, or in the absence of, marked hypertrophy (18). This places a within-species precedent alongside the human carrier data. The precedent concerns functional abnormality preceding hypertrophy; whether atrial abnormality shows the same temporal pattern in genotyped cats is unstudied.

The temporal relationship of atrial to ventricular remodelling deserves particular attention. The conventional model places ventricular pathology first and atrial pathology second, in a strict pressure-driven sequence. Two recent observations complicate that sequence. In the CatScan II cohort, lower left atrial fractional shortening at baseline was an independent predictor of subsequent development of overt HCM in cats whose ventricular wall thicknesses were normal or equivocal at study entry (1). In humans, sarcomere mutation carriers without left ventricular hypertrophy demonstrate atrial structural and functional abnormalities on cardiac magnetic resonance imaging (2). These findings do not establish that atrial pathology causes ventricular hypertrophy. The more parsimonious interpretation is that the disease process expresses itself in the atrium with a sensitivity, and on a timescale, that the conventional ventricle-anchored model does not anticipate. Either way, the atrium cannot be conceptualised as a structure that merely waits for the ventricle to fail.

### 2.3 Functional remodelling: the three atrial roles

Functionally, the left atrium contributes to cardiac output through three sequential roles within each cardiac cycle. As a reservoir during ventricular systole, it accommodates pulmonary venous return against a closed mitral valve, and its compliance during this phase determines pulmonary venous pressure. As a conduit during early ventricular diastole, it transmits blood passively from the pulmonary veins to the relaxing ventricle. As a booster pump during late diastole, atrial contraction completes ventricular filling. Each role is affected differently by the structural changes of feline HCM (14, 15). Reservoir function is compromised early, as atrial wall stiffness rises and compliance falls, with consequences for pulmonary venous pressure that precede overt CHF (11). Conduit function is the most passive of the three roles and, for that reason, the one that depends most on structures other than the atrial wall. Early diastolic transit is driven by the pressure gradient between the atrium and the relaxing ventricle. It therefore depends on the rate and completeness of ventricular relaxation and on ventricular suction, as well as on atrial size, atrial wall stiffness, and the elastic recoil of the atrium (14, 15). In feline HCM these determinants move together: a large, stiff atrium empties passively into a ventricle that relaxes slowly, and the conduit phase carries a smaller share of ventricular filling. Conduit strain thus reflects the atrium and the ventricle jointly, and a reduced value should not be read as an atrial abnormality alone. Booster pump function may be relatively preserved early. It is ultimately blunted by myocyte dysfunction and by arrhythmias, including atrial fibrillation, which where present abolishes coordinated atrial contraction entirely. The clinical importance of this functional taxonomy is that the three roles fail at different rates and produce different consequences. A cat with predominantly impaired reservoir function presents differently from a cat in atrial fibrillation, and the imaging signatures and therapeutic implications differ accordingly. Atrial fibrillation was recorded in only 6 of 1,008 preclinical HCM/HOCM cats in the REVEAL cohort (6). That preclinical denominator will understate prevalence in advanced disease, but the rarity of atrial fibrillation relative to humans nonetheless cautions against uncritical extrapolation of the human construct.

### 2.4 The atrial appendage and arterial thromboembolism

No discussion of atrial pathobiology in feline HCM is complete without specific consideration of the left atrial appendage. The appendage is morphologically and functionally distinct from the body of the atrium. It has greater trabeculation, lower flow velocities under physiologic conditions, and a disproportionate susceptibility to blood stasis as atrial function deteriorates (9, 19). In feline HCM, the appendage is the predominant site of intracardiac thrombus formation, and the embolic events that constitute clinical ATE (saddle thrombus, brachial thromboembolism, renal infarction) are dominated by appendage-derived emboli. Left atrial appendage flow velocity, the presence of spontaneous echo contrast (“smoke”), and the visualisation of frank thrombus are imaging endpoints with direct prognostic and therapeutic implications (19, 20). The appendage is thus the anatomical site at which atrial disease most often produces its catastrophic clinical consequences. Rarer atrial catastrophes, including left atrial tear with haemopericardium, are recognised in practice though not systematically documented in the feline HCM literature.

Stasis is only one limb of Virchow’s triad, and the appendage should be placed within the whole triad rather than treated alone. Hypercoagulability is demonstrable in cats with cardiomyopathy. Stokol and colleagues found laboratory criteria of hypercoagulability in 50% of cats with LA enlargement plus spontaneous echo contrast or thrombus and in 56% of cats with ATE, but in none of the cats with LA enlargement alone, and hypercoagulability was not associated with atrial size or CHF (21). Platelet hyperreactivity adds a cellular component: platelets from cats carrying the MYBPC3 A31P mutation showed increased activation in response to ADP (22), and circulating markers of neutrophil extracellular traps are higher in cats with cardiogenic ATE than in cats without HCM (23). The third limb, endocardial and endothelial injury, is the least directly measured in cats. Higher von Willebrand factor antigen in ATE cats has been interpreted as evidence of endothelial injury (21), and a recent review argues that atrial endocardial damage from wall stress, hypoxia, and inflammation is active well before spontaneous echo contrast appears, so that “smoke” should be treated as a late marker of stasis rather than as the onset of thrombotic risk (24). The appendage therefore remains the site where the triad is completed, but the atrial contribution to ATE is broader than stasis, and risk stratification that waits for echo contrast is stratifying late.

### 2.5 The right atrium and biatrial involvement

The left atrial emphasis of this review reflects the weight of the evidence, not an anatomical claim that disease stops at the interatrial septum. Biatrial enlargement is recognised in advanced feline cardiomyopathy, particularly in restrictive and end-stage phenotypes (8, 9). Right-sided and bicavitary congestive signs are common presentations of feline CHF (9), and tricuspid annular plane systolic excursion carries prognostic information in affected cats (25). Right atrial structure and function in feline HCM remain essentially uncharacterised by modern deformation imaging. The left atrial focus of what follows is a scope boundary set by the available literature, not by the disease.

### 2.6 Implications for the rest of this review

The frame established here (diastolic dysfunction leading to atrial pressure elevation, remodelling, and thence CHF and ATE; Figure 1) is the substrate that the remaining sections trace through imaging, outcomes, therapy, and the consultation.

**Figure 1:**
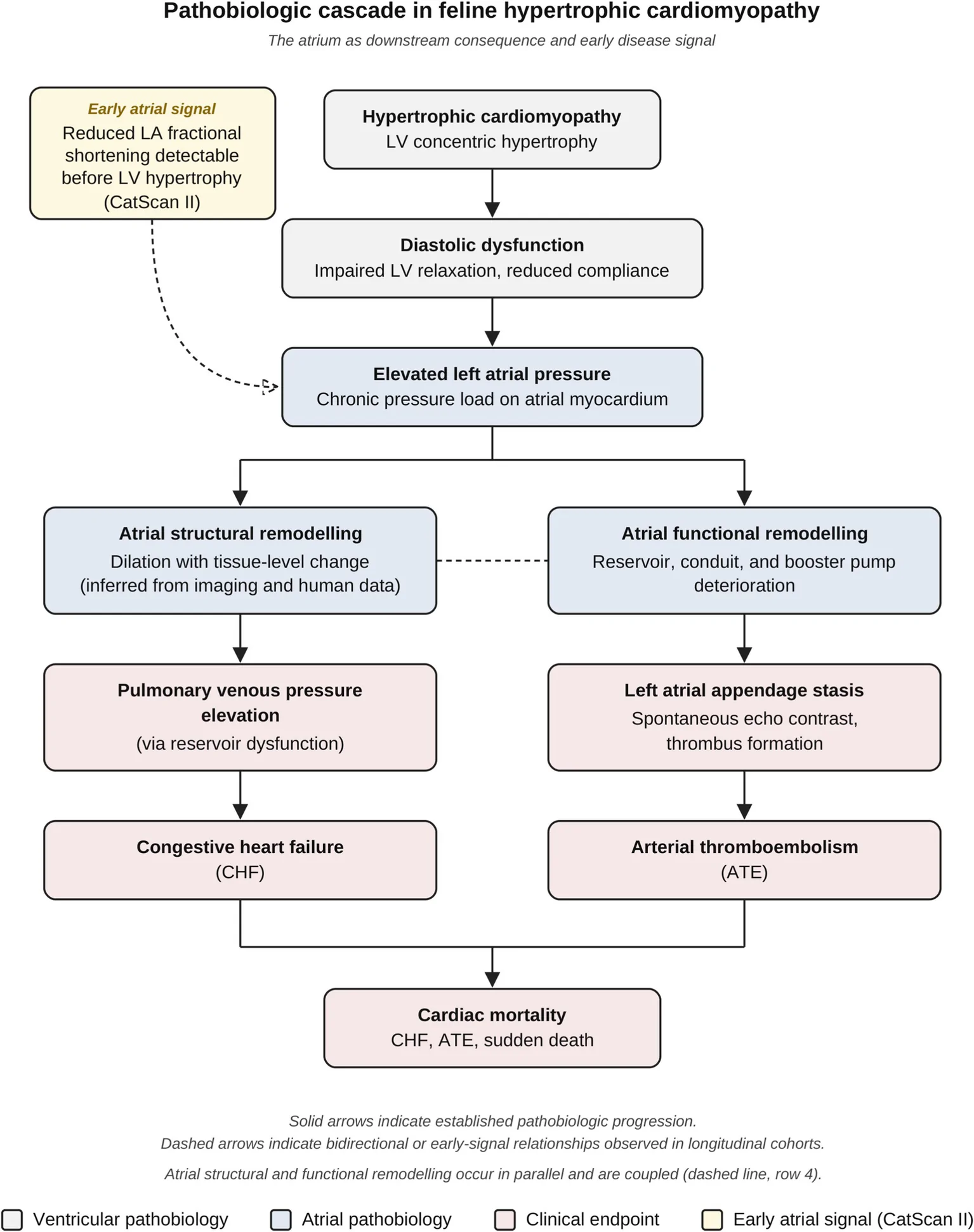
Pathobiologic cascade in feline hypertrophic cardiomyopathy, organised around the left atrium as both downstream consequence and early atrial signal of disease. The conventional pathobiologic sequence proceeds from left ventricular concentric hypertrophy and the associated diastolic dysfunction through chronic elevation of left atrial pressure to atrial structural and functional remodelling, with two clinically dominant endpoints: Pulmonary venous pressure elevation and consequent congestive heart failure (left pathway), and left atrial appendage stasis with consequent thrombus formation and arterial thromboembolism (right pathway). Both endpoints contribute to cardiac mortality. Atrial structural and functional remodelling occur in parallel and are bidirectionally coupled (dashed line, row 4). The early atrial signal annotation (top left, with curved dashed arrow) indicates that reduced left atrial fractional shortening is detectable in cats before the diagnostic threshold for ventricular hypertrophy is met, as demonstrated in the longitudinal CatScan II cohort (1). Solid arrows indicate established pathobiologic progression; dashed arrows indicate bidirectional or early-signal relationships observed in longitudinal cohorts. ATE, arterial thromboembolism; CHF, congestive heart failure; LA, left atrial; LV, left ventricular.

## 3 Imaging the atrium in feline HCM

### 3.1 Atrial size: from M-mode to volumetric

The longest-standing imaging assessment of the left atrium in feline HCM is measurement of size (Table 1). It began with the M-mode left atrium-to-aortic root ratio (LA:Ao), which is rapid, reproducible, and independent of body weight over a wide range (26, 27), and extended to two-dimensional maximum diameter and area at end-systole (27, 28). Schober and colleagues operationalised echocardiographic LA enlargement in cats as a maximum dimension above 1.57 cm or a maximum area above 2.75 cm^2^. Both were measured in the right parasternal long-axis four-chamber view, with discrimination limits derived from healthy cats, and the thresholds remain commonly cited (28). More recent work has extended size assessment to volumetric methods, with single-plane and biplane techniques and three-dimensional volume estimation gaining traction in feline cardiology (29, 30). Volume-based indices are theoretically more robust to atrial geometry than diameter or area alone, and they more closely parallel contemporary human cardiology recommendations (31). The timing of the measurement within the cardiac cycle also matters. Duler and colleagues compared seven LA size and volume measures in 162 cats and found that the minimum LA:Ao ratio (LA:Aomin, measured at end-diastole when the atrium is smallest) and the minimum LA volume from the left apical view best separated cardiomyopathy without CHF from cardiomyopathy with CHF. An LA:Aomin of 1.64 identified CHF with 84% sensitivity and 75% specificity, and LA volumes were not superior to linear measurements (29). Minimum dimensions outperformed maximum dimensions in that study, which is consistent with the atrial argument of this review: the smallest size the atrium reaches after emptying depends on how well it empties, so a minimum dimension is partly a functional measure (Section 3.2). The methodological limitations of size-based assessment are well recognised. They include load dependence, geometric assumptions in non-spherical atria, and beat-to-beat variability. A chronically remodelled atrium may also reach a stable diameter while continuing to deteriorate functionally.

**Table 1:** Echocardiographic modalities for assessment of left atrial structure and function in cats with hypertrophic cardiomyopathy.

Domain	Modality/index (view)	What it measures	Key feline thresholds / reference values	Strengths	Limitations	References
Size	LA:Ao ratio (RPSAx at the aortic valve; M-mode and 2D; maximum and minimum)	Ratio of LA dimension to aortic root	Healthy 2D LA:Ao 1.44 ± 0.28 and median 1.25 (IQR 1.15–1.44) vs. 1.92 ± 0.44 and 2.19 (1.84–2.98) in HCM (10, 11). The ACVIM consensus defines stage B2 qualitatively as moderate to severe LA enlargement and gives no numeric cut-off (9). Maximum LA:Ao 1.96 detected CHF (AUC 0.91) (42). Minimum LA:Ao 1.64 separated cardiomyopathy with CHF from without (sensitivity 84%, specificity 75%) (29). M-mode values run lower than 2D and are not interchangeable (27).	Rapid; low equipment demand; body-weight independent; minimum ratio adds functional information	One-dimensional; load-dependent; timing in cardiac cycle changes the value; ratio originally derived in dogs	(9–11, 26, 27, 29, 42)
Size	2D LA dimension and area (RPLAx four-chamber, last frame before MV opening)	Maximum LA diameter and end-systolic area	Enlargement: LA diameter >1.57 cm or area >2.75 cm^2^, limits derived from healthy cats (28). Healthy LA diameter 12.2 ± 1.4 mm vs. 15.6 ± 2.8 mm in HCM (10). LA diameter 18.5 mm detected CHF (AUC 0.89) (42). Cardiac death hazard rises with diameter: HR 2.58 for 16.1–21.0 mm and 10.73 for ≥21.1 mm vs. ≤16.0 mm (univariable) (5).	Standardised view; widely available; reproducible; outcome-linked	Geometric assumptions; load-dependent; underestimates volume	(5, 9, 10, 27, 28, 42)
Size	LA volume (monoplane or biplane Simpson; 3D)	End-systolic (maximum) and end-diastolic (minimum) atrial volume	Healthy maximum LA volume 1.96 ± 0.54 mL by monoplane or biplane Simpson from the right parasternal four-chamber or left apical two-chamber view, not affected by body weight or age; left apical four-chamber and 3D methods give lower values (30). Minimum volumes discriminated CHF better than maximum volumes, but volumes were not superior to linear measures (29). No indexed (mL/kg) feline reference interval is established.	Robust to geometry; aligns with human standards	Technically demanding; view-dependent; limited feline normative data	(29–31)
Function	LA fractional shortening (LA-FS; M-mode RPSAx or 2D RPLAx)	Percent change in LA dimension from maximum to minimum	Healthy 29.4 ± 4.5% (M-mode) and 29.4 ± 5.5% (2D); HCM stage B 24.7 ± 5.4%; stage C 11.3 ± 5.0%; the two views are not interchangeable (32). Healthy 27.3 ± 6.9% vs. 15.0 ± 8.2% in HCM (10). LA-FS ≤ 25% at baseline predicted later development of HCM, and >25% was associated with 91% lower risk (1). Each 1% increase lowered cardiac death hazard (multivariable HR 0.89) (5).	Simple; reproducible; universally available; outcome-linked	One-dimensional; load-dependent; insensitive to early dysfunction	(1, 5, 7, 10, 32)
Function	LA ejection fraction (LA-EF) and fractional area change (FAC), 2D left apical four-chamber	Percent change in LA volume or area between maximum and minimum	Healthy LA-EF 69.1 ± 13.6% vs. 46.1 ± 21.5% in HCM; FAC 63.7 ± 11.3% vs. 35.5 ± 18.1% (10). Healthy LA-EF 59.2 ± 9.7% vs. 49.4 ± 16.9% in a second cohort (34). LA-EF ≤ 30%: univariable HR 9.72 for cardiac death (5). LA-EF correlates with PALS (*r* = 0.797) and FAC with PALS (*r* = 0.746) (10).	Two-dimensional; well validated against strain	Geometric assumptions; load-dependent; off-axis error	(5, 7, 10, 34)
Mechanics	LA phasic strain by 2D-STE: reservoir (PALS), conduit, contraction	Phase-specific atrial deformation across the cardiac cycle	PALS healthy 28.54 ± 10.31% vs. 13.16 ± 8.64% in HCM (*p* < 0.001); intra-observer 4.7%, inter-observer 14% (statistic not specified) (10). Reservoir strain 21.9 ± 9.5% vs. 14.9 ± 8.7% (34). Median reservoir 21.4% vs. 3.2%, conduit −12.9% vs. −1.5%, contraction −8.6% vs. −1.5% (11). No consensus feline cut-offs; values are vendor-specific.	Sensitive to early dysfunction; separates the three atrial phases	Vendor-specific algorithms; frame rate ≥80 fps; operator-dependent; limited multicentre replication; contraction strain lost in atrial fibrillation	(10, 11, 34)
Mechanics	LA stiffness (LASt)	Ratio of E/e′ to reservoir strain; load-adjusted composite	Median 0.30 (IQR 0.26–0.37) in healthy cats vs. 2.26 (1.45–4.95) in HCM, an approximately sevenfold increase (11). No outcome-derived cut-off; prognostic validation outstanding.	Combines pressure inference with deformation; partially load-adjusted	Inherits error of both inputs; single-cohort feline data	(11, 36)
Adjunctive	TDI at the MV annulus (e′, a′, E/e′)	Annular velocities and non-invasive estimation of LV filling pressure	E/e′ median 6.5 (IQR 5.1–7.4) healthy vs. 7.8 (6.5–15.5) in HCM (11). Lateral e′ > 7.2 cm/s separated normal from HCM (sensitivity 92%, specificity 87%) (39). With E/A fusion at high heart rates, a fused (E + A)/(e′ + a′) ratio >15.1 detected CHF (AUC 0.74), whereas E/e′ alone did not perform well (42). Validated against invasive measurement in cats (37, 38).	Quantitative inference of atrial loading; complements other indices	Annular site influences values; E/A and e′/a′ fusion at feline heart rates; sedation alters values	(11, 37–39, 42)
Adjunctive	Pulmonary venous flow (PW Doppler in a pulmonary vein; S, D, and AR waves)	Venous inflow pattern shaped by LA pressure, compliance, and contraction	Median AR duration 56, 59, and 70 ms in healthy, preclinical HCM, and CHF cats; systolic-to-diastolic dominance shifts toward diastolic in CHF; less useful than transmitral flow and LA size for detecting CHF (42). Peak S velocity correlated with the relaxation time constant, but no pulmonary venous variable correlated with end-diastolic pressure in anaesthetised cats (37). No feline cut-off established.	Interrogates the atrium from the venous side; independent of the mitral inflow signal	S/D fusion at feline heart rates; small vein and difficult sampling; weak evidence for filling-pressure estimation in cats	(37, 41, 42)
Adjunctive	MAPSE / TAPSE (anatomic M-mode, left apical four-chamber)	Long-axis annular plane systolic excursion (LV / RV)	Median MAPSE (free wall) 5.9, 4.7, and 2.8 mm and TAPSE 8.6, 7.2, and 4.6 mm in healthy, asymptomatic HCM, and CHF cats; lowest tertile (MAPSE <3.3 mm, TAPSE <5.3 mm) had shorter survival on univariable analysis (40). In a 184-cat cohort neither index independently predicted outcome (25).	Easy to acquire; reproducible	Indirect index of the atrium; influenced by chamber geometry; no independent prognostic cut-off	(25, 40)
Adjunctive	LAA flow velocity (PW Doppler at the LAA orifice, transthoracic)	Peak outflow velocity at the LAA	Healthy maximum LAA velocity 0.47 ± 0.20 m/s (range 0.24–1.00) (43). Myocardial disease median 0.28 m/s; LAA max <0.20 m/s the only independent predictor of SEC (OR 30.1, 95% CI 4.1–222.3; AUC 0.88); <0.25 m/s increases sensitivity at the cost of specificity (19).	Directly assesses thromboembolic risk; actionable threshold	Operator-dependent; signal quality variable in stressed cats	(19, 43)
Adjunctive	LA spontaneous echo contrast and thrombus (visual)	Qualitative identification of smoke, sludge, or frank thrombus	Presence indicates high ATE risk and is an antithrombotic decision point (9, 19). Laboratory hypercoagulability was found in 50% of cats with LA enlargement plus SEC or thrombus and in none with LA enlargement alone (21). NLR median 8.62 with SEC or thrombus vs. 2.1 without (54).	Direct visualisation of the risk substrate; clinically decisive	Subjective; gain-dependent; varies with imaging plane; a late marker of stasis (24)	(9, 19, 21, 24, 54)

Indices are grouped by what they measure (size, function, mechanics, adjunctive). Values are given as reported in the cited feline studies (mean ± SD or median with interquartile range); populations, views, and equipment differ between studies and the values are not interchangeable across platforms. Where no outcome-derived cut-off exists, this is stated. AR, pulmonary venous atrial reversal wave; ATE, arterial thromboembolism; AUC, area under the receiver operating characteristic curve; CHF, congestive heart failure; HR, hazard ratio; LA, left atrium; LAA, left atrial appendage; LV, left ventricle; MV, mitral valve; NLR, neutrophil-to-lymphocyte ratio; OR, odds ratio; PALS, peak atrial longitudinal strain; PW, pulsed wave; RPLAx, right parasternal long axis; RPSAx, right parasternal short axis; RV, right ventricle; SEC, spontaneous echocardiographic contrast; STE, speckle-tracking echocardiography; TDI, tissue Doppler imaging.

### 3.2 Atrial function: fractional shortening, ejection fraction, fractional area change

The next layer of atrial assessment moves from size to function. Left atrial fractional shortening (LA-FS) is measured by M-mode at the heart base. It captures the percentage change in atrial dimension between end-diastole and end-systole and is the simplest one-dimensional index of atrial contractile performance (7). In healthy cats LA-FS is approximately 29% whether measured by M-mode in the short-axis view or by 2D in the long-axis view, but the two views are not interchangeable in the individual cat and should not be mixed in serial studies (32). Left atrial ejection fraction (LA-EF) and fractional area change (FAC), measured from 2D images, extend the same conceptual approach into two dimensions and capture more of the atrium’s deformation. These functional indices are technically undemanding, broadly available on contemporary echocardiography platforms, and reproducible in trained hands. Linney et al. demonstrated that LA functional indices distinguished cats with HCM from healthy controls and tracked with diastolic dysfunction independent of LA size (7). The minimum LA:Ao ratio described in Section 3.1 belongs conceptually here as well. Because it is measured at end-diastole, after reservoir, conduit, and booster emptying are complete, it integrates atrial size with atrial emptying, which may explain why it discriminated CHF better than the maximum ratio did (29). These indices are load-dependent and one- or two-dimensional, so they cannot characterise atrial mechanics in detail. They are nonetheless the most accessible bridge between atrial size and atrial mechanics for the practising clinician, and they should be part of the standard echocardiographic assessment of any cat in whom HCM is suspected or confirmed.

### 3.3 Atrial mechanics: speckle-tracking strain

Two-dimensional speckle-tracking echocardiography (2D-STE) extends atrial assessment beyond fractional measures to quantify deformation across the cardiac cycle. It generates three physiologically distinct strain components. Peak atrial longitudinal strain (PALS, also termed reservoir strain) reflects reservoir function during ventricular systole. Conduit strain reflects passive transit during early ventricular diastole. Contraction strain reflects active atrial booster pump function during late diastole (14, 15). In feline HCM, Kiatsilapanan and Surachetpong reported PALS values of 13.16 ± 8.64% in HCM cats compared with 28.54 ± 10.31% in healthy controls (*p* < 0.001). Intra- and inter-observer reproducibility for PALS were 4.7 and 14% respectively; the statistic is not further specified in the source (10). Regional analysis showed that the LA roof had the lowest PALS values in HCM cats, with septal and lateral regions also significantly reduced. PALS correlated strongly with conventional indices of atrial function in the same cohort (LA-EF *r* = 0.797, FAC *r* = 0.746, LA-FS *r* = 0.538), suggesting that strain captures a coherent functional signal rather than a noise-driven artefact. Subsequent work compared strain values in HCM cats with and without obstruction (33), and primary research published in 2026 reported global longitudinal, radial, and circumferential strains alongside LA reservoir strain in a larger cohort (34). The methodologic constraints of feline LA strain are familiar from the human and canine literature. Vendor-specific tracking algorithms produce non-interchangeable values, frame rates below approximately 80 frames per second compromise tracking quality, and high heart rates in stressed feline patients challenge boundary identification. Most feline LA strain values published to date originate from a small number of centres. Independent replication across geographic sites and equipment platforms is therefore a methodological gap rather than a methodological strength.

### 3.4 Atrial stiffness as a load-adjusted index

Left atrial stiffness (LASt) is calculated as the ratio of E/e’ (the early diastolic transmitral-to-mitral annular velocity ratio) to reservoir strain. It combines a Doppler-derived index of left ventricular filling pressure with a strain-derived index of atrial deformation, generating a composite index that is partially adjusted for loading conditions (11, 35). LASt has been used in human cardiology as a prognostic marker in heart failure and HCM, and emerging veterinary applications include prognostic work in canine myxomatous mitral valve disease (36). The first feline study, by Tohthong and colleagues, demonstrated significantly elevated LASt in cats with HCM compared with healthy controls, with significant correlations with established echocardiographic indices of atrial dysfunction and elevated filling pressures (11). The conceptual advantage of LASt over strain alone is that it interprets atrial deformation against the loading conditions producing it. This advantage is also its principal limitation: a load-adjusted index inherits the measurement error of both inputs. The feline LASt evidence base remains a single-cohort, single-centre demonstration, and prognostic validation against clinical outcomes is outstanding.

### 3.5 Adjunctive measures: tissue Doppler, pulmonary venous flow, MAPSE, atrial appendage flow, and spontaneous echo contrast

Atrial assessment in feline HCM is complemented by a set of measurements that quantify the structures and flows immediately adjacent to the atrium itself. Tissue Doppler imaging at the mitral annulus quantifies early (e′) and late (a′) diastolic annular velocities. The ratio of transmitral E to annular e′ (E/e′) serves as a non-invasive estimate of left atrial pressure and has been validated against invasive measurement in cats (37, 38). In one series, a lateral annular e’ above 7.2 cm/s separated normal cats from cats with HCM with 92% sensitivity and 87% specificity (39). Mitral and tricuspid annular plane systolic excursion (MAPSE and TAPSE) carry prognostic value in feline HCM, with indirect implications for atrial pressure (25, 40).

Pulmonary venous flow deserves separate comment, because it interrogates the atrium from the venous side and is shaped by LA pressure and compliance more directly than transmitral flow is (41). Pulsed-wave Doppler in a pulmonary vein yields a systolic wave (S) generated by atrial relaxation and mitral annular descent, a diastolic wave (D) that mirrors early ventricular filling, and a reversal wave (AR) produced by atrial contraction against the venous bed. In principle, a blunted S wave with a dominant D wave marks rising LA pressure, and an AR duration that exceeds the transmitral A duration marks elevated ventricular end-diastolic pressure. In cats the technique has real limits. Heart rates of 180–240 per minute fuse S with D and E with A, the vein is small and sample-volume placement is unforgiving, and in the invasive validation study in anaesthetised cats peak systolic pulmonary venous velocity correlated with the relaxation time constant but no pulmonary venous variable correlated with end-diastolic pressure (37). In a clinical HCM cohort, pulmonary venous flow shifted toward diastolic dominance in CHF and AR duration lengthened, but the pulmonary venous variables were less useful for detecting CHF than transmitral flow and LA size (42). We therefore regard pulmonary venous flow as an informative adjunct rather than a core element of the framework. It should be recorded when heart rate and image quality allow, interpreted alongside E/e′ and atrial size rather than alone, and studied prospectively as an atrial endpoint, particularly in sedated or slower-rate cats in which wave separation is feasible.

Most directly relevant to atrial-driven thromboembolic risk are pulsed-wave Doppler interrogation of left atrial appendage outflow velocity (LAA flow velocity) and visual identification of spontaneous echocardiographic contrast and frank thrombus. In healthy cats, maximum LAA flow velocity is approximately 0.47 ± 0.20 m/s (43). Schober and Maerz demonstrated that maximum LAA flow velocity below 0.20 m/s was an independent predictor of left atrial spontaneous echo contrast in cats with myocardial disease (odds ratio 30.1, 95% CI 4.1–222.3). This threshold identifies cats at increased thromboembolic risk (19). The clinical importance of these adjunctive measures is that they convert atrial pathobiology into actionable risk. The cat with an LAA flow velocity below 0.20 m/s and visible spontaneous echo contrast is a different patient from the cat with comparable LA size but preserved appendage flow, and antithrombotic decision-making should reflect that difference.

### 3.6 Reproducibility, limitations, and the replication gap

Three constraints recur across the imaging modalities discussed above. First, much of the recent feline atrial imaging literature, particularly for strain and stiffness indices, originates from a small number of investigators and centres. Independent replication across diverse populations and platforms is incomplete and remains a research priority. Second, vendor-specific tracking algorithms and post-processing pipelines produce non-interchangeable values, which complicates cross-study comparison and the translation of published thresholds to local practice. Third, sedation and anaesthesia influence atrial loading conditions and contractile state, with implications for any imaging study performed under restraint. These constraints argue for a layered atrial assessment in clinical practice (size, function, mechanics, appendage flow) rather than reliance on any single index, and for a research agenda that prioritises multicentre replication and standardisation. Whether these load-dependent indices measure an independent disease participant, or left-sided filling pressure by proxy, is a question taken up directly in the Discussion. The next section traces these imaging substrates through the prognostic and outcomes literature.

## 4 Atrial-driven outcomes in feline HCM

### 4.1 The natural history evidence

Long-term cohort data in feline HCM converge on the finding that the introduction of this review takes as its starting point: atrial parameters carry prognostic information independent of ventricular measurements. The REVEAL study, an international collaborative cohort of cats with preclinical HCM and apparently healthy controls, demonstrated that left atrial enlargement was among the strongest echocardiographic predictors of long-term cardiovascular morbidity and mortality (6). Payne and colleagues, in a referral cohort of 282 cats with HCM, reported 164 deaths, of which 107 were cardiac. Their multivariable model retained left atrial dysfunction as an independent predictor of cardiac death, alongside low left ventricular systolic function and extreme left ventricular hypertrophy (5). The CatScan study established that HCM affects approximately 14.7% of apparently healthy cats screened in rehoming centres. Risk factors included male sex, increasing age, body condition score, and a heart murmur of grade III/VI or louder (3). CatScan II, the longitudinal follow-up cohort, made the most consequential observation for the present review: lower left atrial fractional shortening (25% or below) at baseline was an independent predictor of subsequent development of overt HCM in cats whose ventricular wall thicknesses were normal or equivocal at study entry (1).

### 4.2 Risk stratification: from atrial size to atrial function

The 2020 ACVIM consensus statement on feline cardiomyopathies operationalised left atrial enlargement as the axis for distinguishing stage B1 (low risk of imminent CHF or ATE) from stage B2 (higher risk) (9). The statement also explicitly recognised additional risk factors within stage B2: decreased left atrial function, gallop sound, arrhythmia, extreme LV hypertrophy, LV systolic dysfunction, and spontaneous echo contrast or thrombus (9). The inclusion of “decreased left atrial function” as a B2 modifier is significant. It acknowledges atrial functional impairment as a contributor to risk, but it positions that contribution as a secondary descriptor of an atrium that is already enlarged. The contemporary literature suggests a different sequence is possible. CatScan II identified atrial functional impairment before the LV hypertrophy diagnosis was established (1). Speckle-tracking studies have documented reduced PALS in HCM cats with normal or only modestly enlarged atria (10), and emerging atrial stiffness data extend this earlier-detection capability further (11). The implication is not that the ACVIM staging system is wrong. It is that the atrial dimension on which staging depends should evolve from “how big is the atrium” to “how well does the atrium work.” This evolution has begun in human cardiology with a staged classification of atrial cardiomyopathy (stages 1–3) that integrates biomarkers, atrial geometry, and electrophysiological change (44). Veterinary cardiology has the data to make a similar move, and the disease-modifying era arguably forces it.

### 4.3 Arterial thromboembolism: the lethal endpoint

In the largest international longitudinal cohort of preclinical HCM/HOCM cats, arterial thromboembolism occurred in 11.6% of cats over the observed study period (a crude proportion), with a Kaplan–Meier estimated risk at 10 years after diagnosis of 11.3% (Table 5 of that report) (6). This figure derives from preclinical disease; risk rises once CHF or marked atrial enlargement is established (5, 9). Clinical cohorts document the severity of the event: high mortality, frequent euthanasia at presentation, and substantial disability and recurrence risk among survivors (45, 46). ATE is, in proximate pathophysiology, an atrial event. The thrombus arises in the remodelled left atrium, most often in the appendage, where stagnant blood flow, endocardial dysfunction, and concomitant hypercoagulability complete the Virchow triad (Section 2.4) (19, 21). The imaging risk markers are atrial: severe LA enlargement, low LAA flow velocity, visible spontaneous echo contrast, and frank thrombus, with the LAA flow-velocity threshold detailed in Section 3 (19). Functional imaging adds incrementally to risk stratification; reduced LA strain values have been demonstrated in cats with cardiogenic ATE compared with non-cardiogenic comparators (20). Antithrombotic therapy is similarly atrium-targeted in its rationale. The FAT CAT trial demonstrated clopidogrel to be superior to aspirin for secondary prevention (47), and the ACVIM consensus recommends clopidogrel for at-risk cats, with the addition of a factor Xa inhibitor in highest-risk or post-event cases (9). ATE is not an atrial complication. It is the disease’s atrial endpoint.

### 4.4 Biomarkers in the atrial axis

Circulating biomarkers reflect different aspects of the atrium-ventricle axis. N-terminal pro-B-type natriuretic peptide (NT-proBNP) is released mainly from ventricular cardiomyocytes in response to wall stress and volume loading. It responds to atrial stretch indirectly: elevated LA pressure is the consequence of the ventricular filling pressure that drives ventricular wall stress, and the atrial contribution to circulating concentrations grows only as atrial stretch becomes marked (48, 49). NT-proBNP has demonstrated screening utility in feline cardiomyopathy and prognostic value in established disease. It is not an atrial-specific marker, but its behaviour in feline HCM is dominated by the atrial axis once LA pressure becomes elevated (6, 50). Atrial natriuretic peptide (ANP) is, by contrast, released directly from atrial myocytes in response to atrial stretch and is the more atrial-specific of the two peptides. In a prospective multicentre study of 161 cats, plasma ANP was not elevated in cats with cardiomyopathy without LA dilation, but it rose with LA dilation and further with heart failure; a cut-off above 110.9 pg/mL identified LA dilation with 73.6% sensitivity and 93.5% specificity (AUC 0.86) (51). NT-proANP behaves similarly, although in a direct comparison NT-proBNP discriminated cats with heart disease from controls better (52). ANP assays are less widely available in veterinary practice than NT-proBNP, and neither peptide has been evaluated as a longitudinal atrial endpoint. The direct atrial origin of ANP nevertheless makes it a natural candidate biomarker for atrial-focused trials. Cardiac troponin I (cTnI) reflects myocardial injury more broadly, with an LV bias in HCM but with elevations that track disease severity and prognosis (53). In the disease-modifying era, these biomarkers acquire an additional role: serial measurement may inform response to therapy, an application that will be considered in Sections 5 and 6.

A haematologic index also belongs in the atrial axis. The neutrophil-to-lymphocyte ratio (NLR), obtainable from any complete blood count, was higher in HCM cats with CHF, was associated with LA size and appendage function, and was markedly higher in cats with LA spontaneous echo contrast or thrombus (median 8.62) than in HCM cats without (2.1); cats in the highest NLR tertile had a hazard ratio for cardiac death of 10.26 (54). A second study found the highest NLR in cats with cardiogenic ATE, and reported that a low platelet-to-neutrophil ratio predicted cardiac death (55). NLR is not atrial-specific and is moved by stress, glucocorticoids, and intercurrent disease. Where echocardiography is not affordable, however, it is an inexpensive marker that tracks the thrombogenic atrium, and it can flag the cat in whom appendage interrogation should not be deferred.

## 5 The therapeutic reframe in the disease-modifying era

Atrial parameters carry independent prognostic information in feline HCM; the ventricle defines the disease’s structural identity. That asymmetry frames this section.

### 5.1 RAPACAT and the conditional approval landscape

RAPACAT was a multicentre, placebo-controlled, double-blinded trial. It randomised 43 client-owned cats with subclinical, non-obstructive HCM to one of three arms: placebo, delayed-release rapamycin at 0.3 mg/kg once weekly, or delayed-release rapamycin at 0.6 mg/kg once weekly (13). Exclusions restricted the population to ACVIM stage B1 or B2 subclinical disease without severe outflow-tract obstruction. The primary outcome was maximum LV wall thickness at any location; 36 cats completed all visits. At day 180, low-dose delayed-release rapamycin significantly reduced maximum wall thickness compared with placebo (*p* = 0.01, against an unadjusted alpha of 0.10 prespecified for this exploratory, dose-determining trial). The corresponding day 60 comparison did not reach significance (13). The high-dose group did not show a significant effect versus placebo. The investigators attributed this either to possible type II error, with only 10 high-dose cats evaluable at day 180, or to a more favourable balance of mTORC1 suppression and mTORC2 sparing at the lower weekly dose (13). Serious adverse events were numerically more frequent with rapamycin (4 versus 1; not statistically significant). They included one death from diabetic ketoacidosis in an obese low-dose cat with pre-existing diabetic risk factors, and the label’s contraindications for diabetic and hepatically compromised cats reflect these considerations (13, 56). On the strength of these results, the FDA granted conditional approval to Felycin-CA1 in March 2025 for management of ventricular hypertrophy in cats with subclinical HCM (12). Conditional approval means that a reasonable expectation of effectiveness has been shown while a full demonstration of effectiveness is still pending (12, 56). Veterinary cardiology now has its first conditionally approved disease-modifying drug for feline HCM, and the qualifier matters.

Several qualifications follow, and they are stated here so that the remainder of this review is not read as an endorsement. First, RAPACAT is the only published randomised trial of rapamycin in cats with HCM. A single 180-day trial of 43 cats with 36 completers, an exploratory alpha, and a significant result at only one dose and one time point cannot establish efficacy. It establishes a hypothesis, which the HALT HCM trial (Section 5.5) is designed to test. Second, rapamycin is not a selective mTORC1 inhibitor. Chronic exposure also disrupts mTORC2, which regulates insulin signalling, glucose homeostasis, and lymphocyte function, and hyperglycaemia, new-onset diabetes mellitus, and immunosuppression are recognised class effects in other species (57). The product label contraindicates use in cats with diabetes mellitus or pre-existing liver disease, warns that the drug may impair the response to vaccination (only the response to a killed rabies vaccine was evaluated), lists cytochrome P450 3A4 and P-glycoprotein interactions, and states that safety has not been evaluated in cats with chronic kidney disease, hyperthyroidism, or chronic viral infection, or in cats receiving beta-blockers or corticosteroids (56). Third, the approval is a United States conditional approval only. To the authors’ knowledge, no veterinary sirolimus formulation is registered for cats in Europe or on other continents at the time of writing. Human sirolimus formulations and compounded products have not been tested for pharmacokinetics, tolerability, or efficacy in cats with HCM, and the delayed-release matrix of the approved product is part of the tested dosing regimen. Extrapolation of the RAPACAT dose to such products is therefore unsupported by evidence, and the authors do not recommend it. The clinician outside the United States has a published trial but no product, and the clinician inside it has a product whose evidentiary basis is provisional.

### 5.2 What was measured beyond the ventricle

Three observations from the RAPACAT design are relevant here. First, maximum left atrial diameter was tracked as a secondary echocardiographic outcome at all on-treatment time points (13). The atrium was present in the trial, but as a unidimensional structural measurement. Second, an exploratory analysis correlated higher baseline NT-proBNP with faster subsequent wall-thickness progression (13); because NT-proBNP reflects ventricular wall stress directly and responds to atrial stretch only indirectly, through the filling pressure that both chambers share (Section 4.4), cats whose atria were already responding to load may have progressed faster. Third, atrial functional indices were not part of the trial’s outcome assessment. Left atrial fractional shortening, left atrial ejection fraction, fractional area change, peak atrial longitudinal strain, and atrial stiffness were not reported as primary or secondary endpoints. The atrium was sized, but its mechanics were not characterised. The evidentiary basis for the conditional approval therefore rests on a ventricular structural endpoint, a unidimensional atrial structural endpoint, and biomarker data whose atrial component can be partly inferred but was not directly measured.

### 5.3 The mechanistic question through an atrial lens

The mechanistic basis for the RAPACAT result is well established. Selective mTOR inhibition prevents and reverses pathological cardiomyocyte hypertrophy in rodent disease models, and observational data from human renal allograft recipients treated with sirolimus report reductions in left ventricular wall thickness (57, 58). The mTOR pathway is implicated in the progression of feline HCM at the molecular level (13). Whether the same pathway drives atrial remodelling in feline HCM, with the same magnitude and direction of effect, is a separate empirical question. Atrial and ventricular cardiomyocytes differ in their fundamental transcriptional and contractile programmes, and atrial fibroblasts respond differently to profibrotic stimuli than ventricular fibroblasts do (17, 59). In humans, contemporary atrial cardiomyopathy biology distinguishes atrial-selective fibrotic remodelling that may proceed independently of ventricular pathology (17, 44). Whether mTOR inhibition in cats reverses atrial fibrosis (if it occurs in cats as it does in humans), restores atrial reservoir or contraction strain, normalises atrial stiffness, or alters appendage flow has not been answered by the published RAPACAT data. The clinical translation that matters most to the cat with HCM is whether disease-modifying therapy alters the atrial trajectory, because the atrial trajectory drives CHF and ATE. The mechanistic assumption that ventricular hypertrophy reduction will translate to atrial benefit is plausible but unverified.

A related question is whether mTOR inhibition could harm an atrium that is already functionally impaired while the ventricle remains hypercontractile. mTORC2 supports contractile function of the pressure-loaded mouse heart, and its loss unmasks dysfunction under load (60). If chronic rapamycin exposure reduces mTORC2 activity in atrial myocardium, the atrium, which in HCM is the chamber under sustained load and with the smaller functional reserve, would be the chamber most exposed. The opposite direction is also plausible: in an aged-mouse model, rapamycin attenuated atrial structural and electrical remodelling and reduced atrial fibrillation inducibility (61). Neither direction has been examined in cats, and RAPACAT measured no atrial functional index. We regard an adverse atrial effect as unproven but not excluded, and this uncertainty is an additional reason to include atrial functional endpoints in HALT HCM and its successors. One atrial-relevant effect has been examined ex vivo. In healthy cats dosed with low-dose delayed-release rapamycin for 4 weeks, platelet aggregation was unchanged, but P-selectin expression and markers of the procoagulant platelet phenotype were reduced (62). Whether this translates into a lower thrombotic tendency in cats with a remodelled atrium is unknown, and it should not be read as a reason to withhold antithrombotic therapy (Section 5.4). It does, however, identify platelet function as an inexpensive atrial-axis endpoint that future trials could measure, and it opens a research direction in which disease-modifying and antithrombotic effects might be examined in the same cat.

### 5.4 Antithrombotic therapy as the existing atrium-targeted intervention

The contemporary feline HCM treatment armamentarium contains one therapeutic category whose rationale is unambiguously atrial: antithrombotic therapy. The FAT CAT trial established clopidogrel as superior to aspirin for secondary prevention of cardiogenic ATE (47), and the 2020 ACVIM consensus recommends clopidogrel for cats at risk of ATE, with the addition of a factor Xa inhibitor in highest-risk or post-event cases (9). The prescribing rationale rests on the atrial pathobiology established earlier in this review. Stagnant blood in a remodelled, dysfunctional left atrium, particularly in the appendage, creates the substrate for thrombus formation. Antithrombotic decision-making is fundamentally atrium-driven, even where prescribers do not frame it that way. The arrival of disease-modifying therapy does not alter the antithrombotic indication or evidence base. A cat with significant atrial enlargement, low LAA flow velocity, or visible spontaneous echo contrast remains an antithrombotic candidate whether or not it is also receiving Felycin-CA1. Disease-modifying therapy aimed at the ventricle should not be misconstrued as reducing the cat’s atrial-driven thromboembolic risk.

In humans, the 2024 guideline treats clinical atrial fibrillation in HCM as a Class 1 indication for anticoagulation independent of conventional stroke scores, recognising the remodelled HCM atrium as itself thrombogenic. The parallel with atrium-anchored antithrombotic reasoning in cats is direct (4).

### 5.5 HALT HCM and the case for atrial endpoints in future trials

The HALT HCM trial, sponsored by TriviumVet and currently enrolling at over 20 veterinary centres in the United States, aims to enrol approximately 300 cats with subclinical HCM into a 12-month, blinded, randomised, placebo-controlled study with five post-enrolment visits (63). The trial is several-fold larger than RAPACAT and substantially longer in dosing and follow-up. The primary efficacy endpoint has not been specified in the publicly available trial information, and no atrial functional endpoint is described there. If HALT HCM reproduces the RAPACAT result, the case for Felycin-CA1 in subclinical feline HCM will be strengthened on its current evidentiary axis. On the information currently available, whether the atrial axis will be measured cannot be determined. From the perspective of this review, the more consequential design question is what comes after HALT HCM. Future feline HCM trials should incorporate atrial functional endpoints (fractional shortening, ejection fraction, peak atrial longitudinal strain, atrial stiffness), atrial appendage flow characteristics, and clinically defined atrial-driven outcomes (CHF onset, ATE incidence, all-cause cardiac mortality). The argument is not that ventricular wall thickness is a poor primary endpoint. It is that ventricular wall thickness alone cannot answer the clinical question that disease-modifying therapy is ultimately asked to answer: whether treatment alters the disease’s atrial trajectory and the outcomes that trajectory generates. The next section translates this evidentiary asymmetry into the cardiology consultation.

## 6 Reframing the cardiology consultation: an atrial-centric framework

### 6.1 The atrial-centric workup

The diagnostic workup of a cat with suspected or confirmed HCM, in an atrial-centric frame, comprises a layered assessment (Figure 2) that proceeds from chamber size through atrial function and mechanics to appendage flow and adjunctive measures. The minimum dataset includes left atrial dimension or area indexed to aortic root (M-mode or 2D LA:Ao, recording both the maximum and the minimum ratio where feasible, because the minimum ratio carries functional information and discriminated CHF better than the maximum ratio in one cohort) (29); a left atrial functional measure (LA-FS, LA-EF, or fractional area change) to quantify the atrium’s mechanical response to its loading conditions; and tissue Doppler interrogation at the mitral annulus to estimate filling pressure. These, with appendage interrogation, form the decision-ready tier: measures validated widely enough to inform decisions today. A second tier should be recorded and tracked within the individual patient but not yet applied against published thresholds. Peak atrial longitudinal strain (ideally with conduit and contraction strain) and atrial stiffness add sensitivity to early atrial dysfunction that conventional indices do not match, but their feline evidence base remains single-centre and vendor-specific (10, 11). Pulsed-wave Doppler interrogation of left atrial appendage flow and visual assessment for spontaneous echo contrast or thrombus complete the atrial-imaging dataset. They are clinically essential in any cat in whom thromboembolic risk stratification is in question (19). The workup yields a profile rather than a single number. Serial recording at each consultation establishes the trajectory of the atrium, which is the trajectory of the disease’s clinical risk.

**Figure 2:**
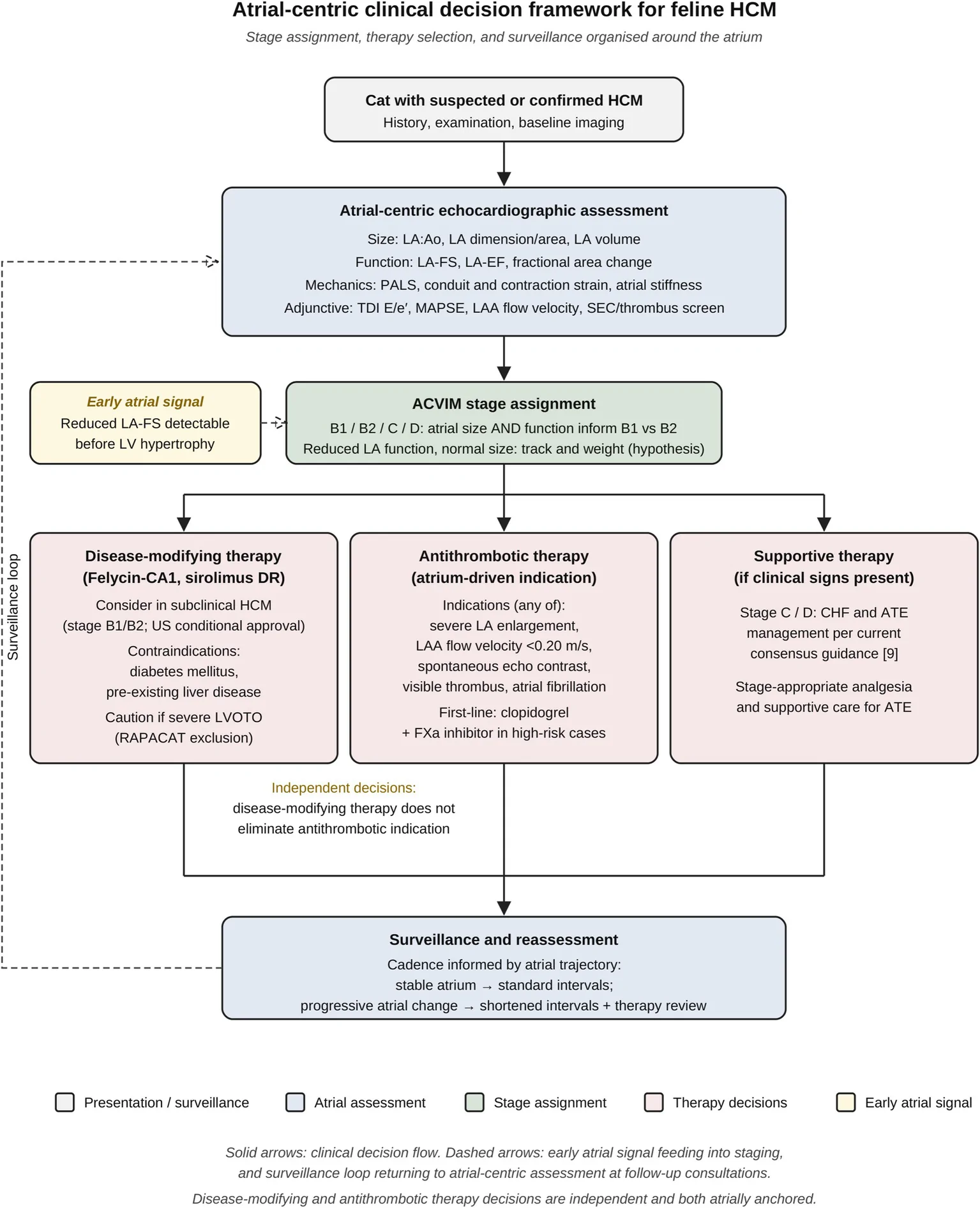
Atrial-centric clinical decision framework for feline hypertrophic cardiomyopathy in the disease-modifying era. The cardiology consultation begins with presentation (history, examination, baseline imaging) and proceeds through a layered atrial-centric echocardiographic assessment that includes atrial size (LA:Ao ratio, dimension, area, and volume), atrial function (LA fractional shortening, ejection fraction, and fractional area change), atrial mechanics (peak atrial longitudinal strain, conduit and contraction strain, and atrial stiffness), and adjunctive measures (tissue Doppler at the mitral annulus, mitral and tricuspid annular plane systolic excursion, left atrial appendage flow velocity, and screening for spontaneous echo contrast or thrombus). Stage assignment within the 2020 ACVIM consensus framework is informed by atrial size and atrial function in combination, with reduced atrial function in the absence of atrial enlargement recorded and tracked as a hypothesis for prospective testing rather than as a reclassification. The early atrial signal annotation (left side) indicates that reduced left atrial fractional shortening can be detectable before the diagnostic threshold for left ventricular hypertrophy is met, as demonstrated in the CatScan II longitudinal cohort (1). Three therapy decision domains follow stage assignment. Disease-modifying therapy (Felycin-CA1, delayed-release sirolimus) may be considered for cats with subclinical HCM (stage B1 or B2) meeting the labelled criteria, on the basis of a United States conditional approval supported by a single exploratory trial (Section 5.1), with contraindications for diabetes mellitus and pre-existing liver disease, label warnings on vaccine response and drug interactions, and prescribing prudence in obstructive disease pending further evidence. Antithrombotic therapy is an atrium-driven decision based on imaging-defined risk markers (severe LA enlargement, low LAA flow velocity, spontaneous echo contrast or thrombus, atrial fibrillation), with clopidogrel as first-line and addition of a factor Xa inhibitor in highest-risk cases. Supportive therapy is initiated when stage C or D clinical signs are present. Disease-modifying and antithrombotic decisions are independent and both atrially anchored: Initiation of disease-modifying therapy aimed at the ventricle does not eliminate the indication for antithrombotic therapy in a cat whose atrium remains at risk. Surveillance cadence is informed by atrial trajectory across consultations; a stable atrium permits standard follow-up intervals, while progressive atrial dilation, declining atrial function, declining atrial strain, or rising atrial stiffness should prompt shortened intervals and therapy reassessment. Solid arrows indicate clinical decision flow; dashed arrows indicate the early atrial signal feeding into staging and the surveillance loop returning to atrial-centric assessment at follow-up consultations. ACVIM, American College of Veterinary Internal Medicine; ATE, arterial thromboembolism; CHF, congestive heart failure; DR, delayed-release; FXa, factor Xa; HCM, hypertrophic cardiomyopathy; LA, left atrial; LA-EF, left atrial ejection fraction; LA-FS, left atrial fractional shortening; LAA, left atrial appendage; LV, left ventricular; LVOTO, left ventricular outflow tract obstruction; MAPSE, mitral annular plane systolic excursion; PALS, peak atrial longitudinal strain; RAPACAT, the Rapamycin in Cats clinical trial; SEC, spontaneous echocardiographic contrast; TDI, tissue Doppler imaging.

### 6.2 Stage-anchored therapy decisions in the disease-modifying era

The 2020 ACVIM staging system remains the appropriate clinical scaffold for therapy decisions in feline HCM, and the atrial-centric framework operates within it rather than displacing it (9). What changes is the texture of the B1 versus B2 distinction. In the conventional reading, B2 is defined primarily by left atrial enlargement, with additional atrial-functional descriptors as risk-modifying factors. In the atrial-centric reading, B1 versus B2 is informed equally by atrial functional impairment and by atrial size. Whether a cat with normal atrial dimensions but reduced LA-FS, reduced peak atrial longitudinal strain, or elevated atrial stiffness should be managed as closer to B2 is a hypothesis this framework generates for prospective testing, not a recommendation it can yet support; the 2020 consensus already lists decreased LA function as a B2 risk modifier (9). The practical difference proposed is deliberate tracking and weighting of atrial function rather than incidental notation (1, 10, 11). Disease-modifying therapy with delayed-release rapamycin (Felycin-CA1) may be considered in stage B1 and B2 cats meeting the labelled criteria, where the product is available and on the understanding that its conditional approval rests on a single exploratory trial and that a full demonstration of effectiveness is still pending. The product label’s contraindications (diabetes mellitus, pre-existing liver disease), its warnings on vaccine response and drug interactions, the trial’s exclusion of severe LVOTO, and the absence of any tested feline formulation outside the United States should all inform that decision (Section 5.1) (12, 13, 56). Owners should be told that the evidence is provisional and that monitoring for hyperglycaemia and hepatic injury is part of treatment. Antithrombotic therapy is a separate decision driven by atrial-centric risk markers (severe LA enlargement, low LAA flow velocity, spontaneous echo contrast, atrial fibrillation), with clopidogrel as first-line and consideration of factor Xa inhibitor addition in highest-risk cases (9, 47). These two decisions are independent. Ventricle-aimed disease-modifying therapy does not eliminate the indication for antithrombotic therapy in a cat whose atrium remains at risk.

### 6.3 Surveillance and progression

Follow-up cadence in feline HCM has historically been informed by the severity of the ventricular phenotype and by clinical signs. Within the atrial-centric framework, the trajectory of atrial parameters provides an additional and arguably more informative basis for surveillance scheduling. Serial atrial comparisons should be interpreted against unchanged loading conditions; diuretic dose, volume status, blood-pressure control, and thyroid status all move atrial indices independently of the disease trajectory. Managed this way, the consultation itself becomes the instrument through which the atrial trajectory is read. A cat whose atrial size and function are stable across consultations may be followed at standard intervals. A cat showing progressive atrial dilation, declining LA-FS, declining peak atrial longitudinal strain, or rising atrial stiffness should prompt shortened intervals and reassessment of therapeutic decisions, whether or not ventricular wall thickness has materially changed (1, 7). Biomarkers complement imaging-based surveillance. NT-proBNP serial measurement may be particularly useful where echocardiographic capacity is limited (48, 49). cTnI adds an injury axis: a concentration above 0.7 ng/mL predicted cardiac death independently of heart failure and LA dilation in one cohort (53), admission and follow-up concentrations predicted survival in another (64), and a rising cTnI in a cat whose atrial indices are also deteriorating is a stronger signal than either alone. ANP, released directly by atrial stretch, is the most atrial-specific serum marker available and would be the logical biomarker for atrial-trajectory surveillance if assay access improves (Section 4.4) (51). The NLR, available from routine haematology, can be tracked at the same visits and is most informative when it rises in a cat with a low appendage flow velocity (Section 4.4) (54). Cats receiving disease-modifying therapy require periodic monitoring of biochemistry, blood glucose, and the relevant safety parameters established by the product label, alongside the cardiologic assessment (56).

### 6.4 Transient myocardial thickening: the atrial lens on a ventricular mimic

Not every thickened feline ventricle is HCM, and the atrium helps to tell the difference. Transient myocardial thickening (TMT) is a syndrome, described by Novo Matos and colleagues, in which cats, typically young, present with LV wall thickening and often CHF after an antecedent event (general anaesthesia, trauma, vaccination, infection, or fever) and then undergo complete reverse remodelling (65). In that series of 21 TMT cats compared with 21 HCM cats, median age was 2 versus 8 years, an antecedent event was identified in 71% versus 29%, wall thickness fell from 6.8 to 4.8 mm over a median of 3.3 months, cardiac medication was discontinued in 20 of 21 TMT cats, and all TMT cats survived, whereas 8 of 19 HCM cats died (65). Cardiac troponin I was high at presentation in TMT (median 2.1 ng/mL) and fell to 0.01 ng/mL on recovery, consistent with myocardial injury or inflammation as the substrate. The difference from HCM cats at presentation (median 1.2 ng/mL) did not reach significance in that small series, so cTnI supports the diagnosis and tracks recovery without discriminating TMT from HCM at the first visit (65). A multicentre series of 27 cats found a median cTnI of 5.5 ng/mL, CHF in 25 of 27, a possible trigger in half, and a median of 43 days to significant reduction in wall thickness (66). A 2025 study found that 16 of 53 cats with an HCM phenotype met TMT criteria, and that two cats initially classified as TMT proved to have HCM with superimposed TMT, so the two conditions can coexist (67).

The atrium is where the difference shows most clearly on follow-up. In the original series, LA:Ao fell from 1.8 to 1.45 in TMT cats while it rose from 2.4 to 2.6 in HCM cats (65), so atrial reverse remodelling accompanied ventricular reverse remodelling. At first presentation, however, the distinction is often impossible on imaging alone. It rests on age, history, cTnI, and serial examination, and no atrial functional or strain data in TMT have been published. Two practical consequences follow. First, a cat diagnosed with HCM whose atrial size and function normalise on serial examination should prompt reconsideration of the diagnosis and of the need for continued treatment, including disease-modifying therapy, which has no evidence base in TMT and would expose a recovering cat to risk without benefit. Second, TMT is a natural experiment in atrial reversibility. Comparing LA-FS, LA strain, and atrial stiffness between TMT and HCM at presentation, and following them through recovery, could show whether atrial mechanics separate the two conditions earlier than wall thickness does. That comparison is added to the research agenda in Section 7.

## 7 Research and practice agenda

The atrial-centric framework proposed in this review carries with it a set of research and practice priorities that follow directly from the gaps identified across Sections 2–6.

Atrial endpoints in clinical trials. The most consequential research priority is the incorporation of validated atrial functional endpoints into feline HCM clinical trials. Future trial designs should include left atrial fractional shortening, peak atrial longitudinal strain, and where feasible atrial stiffness, alongside the established ventricular wall thickness endpoint. They should also specify atrial appendage flow velocity and surveillance for spontaneous echo contrast as separate prespecified outcomes. Where event-driven endpoints are achievable within the trial duration, congestive heart failure onset, arterial thromboembolism incidence, and all-cause cardiac mortality represent the clinical questions that the disease’s atrial trajectory ultimately raises. The same priorities apply to post-marketing surveillance of conditionally approved disease-modifying therapy, where the question of whether ventricular hypertrophy reduction translates to atrial benefit can be answered most directly by measuring the atrium. Endpoint measurement variability bears directly on power. The reported 14% inter-observer variability for peak atrial longitudinal strain (statistic not specified in the source) (10) inflates required sample sizes unless mitigated. Centralised core-laboratory reading, the established mitigation in human imaging trials, belongs in any protocol adopting atrial strain endpoints.

Replication and standardisation. A meaningful share of the contemporary feline atrial mechanics literature originates from a small number of investigators and centres. Independent multicentre replication of left atrial strain and atrial stiffness reference values across diverse populations and equipment platforms is a methodological priority. Standardisation of acquisition protocols, frame rate requirements, and post-processing approaches would support cross-study comparison and translation of published thresholds to local practice. Sedation effects on atrial parameters in feline patients require systematic characterisation, given how often echocardiography is performed under restraint. Systematic histopathologic characterisation of the feline atrial myocardium belongs on the same list; the cross-species analogy invoked in Section 2 remains defensible only while the missing feline histology is actively sought. Genotyped MYBPC3-positive cohorts offer the natural population in which to test the atrial-precursor hypothesis prospectively (18). A comparison of atrial functional and mechanical indices between cats with transient myocardial thickening and cats with HCM, at presentation and through recovery (Section 6.4), is a specific and feasible study that could shorten the time to a correct diagnosis and spare recovering cats unnecessary treatment (65).

Clinical practice infrastructure. Reference values for atrial mechanics stratified by breed, age, body size, and obstructive phenotype are needed to support routine clinical interpretation of strain and stiffness indices. Decision-support thresholds for therapy initiation, particularly for the boundary between stage B1 and B2 informed by atrial functional measures, would translate the framework proposed in this review into specific practice recommendations. Surveillance interval recommendations grounded in longitudinal atrial trajectory data, rather than ventricular phenotype severity alone, would close the gap between available imaging methods and the follow-up cadence that uses them.

## 8 Discussion

This review complements, and is distinct from, recent comprehensive reviews of feline HCM. Kaplan and colleagues reviewed the animal-model and molecular foundations of targeted therapeutics, including the mTOR pathway work that culminated in the RAPACAT trial (68). de Sousa and colleagues provided a broad clinical-diagnostic and therapeutic update organised around the 2020 ACVIM consensus (69). Both are organised primarily around the ventricular phenotype and the therapeutic pipeline. The present review differs in its organising question. It traces a single anatomical structure, the left atrium, across pathobiology, imaging, outcomes, therapy, and the consultation, and asks what changes when the disease is viewed from the chamber that generates its clinically decisive events.

The ventricular anchoring of feline HCM is rational rather than arbitrary. Wall thickness is measurable on any echocardiographic platform, defines the disease in cats as in humans (4, 9), and was a feasible and defensible primary endpoint for a 180-day trial in which event-driven outcomes were unachievable (13). It is, however, a less stable criterion than its universality suggests. Transient myocardial thickening (Section 6.4) and hydration-dependent pseudohypertrophy are both recognised in the consensus differential for ventricular thickening (9), hyperthyroidism and systemic hypertension confound it, the 6 mm cut-point is operational rather than biological, and the measurement is made at millimetre scale on a wall a few millimetres thick. Together these mean the diagnostic anchor is not only late to the disease but imprecise at its boundary (3, 9). The atrial-centric framework proposed here builds on that foundation rather than indicting it. Ventricular hypertrophy remains the diagnostic entry point; the argument of this review concerns what should be measured, staged, and followed once the diagnosis is made.

A second objection deserves engagement at full strength. Every atrial index in this review, from atrial size through fractional shortening, ejection fraction, strain, E/e′, and stiffness, is load-dependent. An atrial-centric framework can therefore be recast as measuring left-sided filling pressure with several instruments rather than one: the atrium as barometer of ventricular diastolic failure rather than as disease participant. Four observations argue against the pure-barometer reading. Atrial functional indices track diastolic dysfunction independently of atrial size, a redundancy the barometer model would not predict (7). The appendage phenomena that drive thromboembolism constitute a thrombogenic substrate with its own morphology and outcome, not a pressure readout (19). Atrial-selective fibroblast biology supplies a mechanism by which atrial remodelling can proceed on its own trajectory (17, 59). And CatScan II documents atrial dysfunction in cats with normal or equivocal ventricular geometry (1). Even where the atrium is partly a barometer, it is positioned closer to the decisive clinical events than wall thickness is, and that alone sustains the trial-endpoint argument.

This review has limitations. It is a narrative rather than a systematic review; no systematic inclusion criteria or risk-of-bias assessment were applied, and the selection of cited literature reflects a judgement of relevance rather than an exhaustive audit. The feline atrial mechanics literature on which several arguments rest is concentrated in a small number of centres and awaits independent multicentre replication, a limitation that Section 7 converts into a research priority. The therapeutic sections depend heavily on a single exploratory, dose-determining trial with 36 completers and an unadjusted alpha of 0.10, and on regulatory documents that may evolve as conditional approval progresses. The authors make no recommendation on the use of delayed-release rapamycin beyond the labelled population in the jurisdiction where it is conditionally approved, and the safety profile summarised in Section 5.1 should be read as a reason for restraint rather than as a footnote. Finally, the consultation framework proposed in Section 6 is a synthesis of existing evidence rather than a validated decision tool; its prognostic performance has not been tested prospectively. The framework also offers no mechanism for sudden cardiac death, which at 2.2% was the least common of the three terminal endpoints in the REVEAL cohort (6). Figure 1 records it as an endpoint without claiming to explain it.

## 9 Conclusion

The clinical events that determine quality and duration of life in cats with HCM are atrial in their proximate pathophysiology. Congestive heart failure arises from elevated left atrial pressure, arterial thromboembolism from stasis in a remodelled left atrial appendage, and survival tracks atrial size and function independently of ventricular wall thickness. Contemporary imaging can quantify atrial structure, function, and mechanics with increasing sensitivity. Yet diagnostic frameworks, prognostic models, and the regulatory endpoint underpinning the first conditionally approved disease-modifying therapy remain anchored on the ventricle. Incorporating atrial functional endpoints into clinical trials, replicating atrial mechanics methodology across centres, and structuring the cardiology consultation around the atrial trajectory are the practical steps that follow. The atrium has been part of the feline HCM evidence base for decades. The proposition of this review is that it should now be at the centre.
